# Secretin-stimulated MR cholangiopancreatography: spectrum of findings in pancreatic diseases

**DOI:** 10.1007/s13244-016-0517-2

**Published:** 2016-09-15

**Authors:** Piero Boraschi, Francescamaria Donati, Rosa Cervelli, Federica Pacciardi

**Affiliations:** Division of Diagnostic and Interventional Radiology-Department of Translational Research and New Technologies in Medicine, University of Pisa, Pisa University Hospital-Via Paradisa 2, 56124 Pisa, Italy

**Keywords:** MR cholangiopancreatography, Secretin, Pancreas, Pancreatic ducts, Pancreatic function

## Abstract

**Abstract:**

MR cholangiopancreatography (MRCP) is an imaging technique that has evolved over the past 2 decades and that continues to have a fundamental role in the non-invasive detection of morphologic features of the pancreatic ducts. In several studies, MRCP has shown a good correlation with endoscopic retrograde cholangiopancreatography in the evaluation of diseases and anatomic variants of the pancreatic ductal system. However, in physiologic conditions the pancreatic ducts are not always easily recognisable. More recently, secretin-enhanced MRCP protocols have been developed for a more complete assessment of pancreatic ducts and glandular function, including monitoring of pancreatic flow dynamics and duodenal filling after pancreatic hormonal stimulation with secretin. The injection of this hormone causes temporary dilation of the pancreatic ducts, principally by increasing pancreatic exocrine secretions, and thus improving MRCP detection of the ducts and characterisation of pancreatic disorders and allowing the assessment of the exocrine pancreatic reserve. The purpose of this pictorial review is to summarise the technical aspects of secretin-stimulated MRCP, to report the secretin-stimulated MRCP findings of pancreatic duct abnormalities and to review the diagnostic capabilities of secretin-stimulated MRCP in various pancreatic ductal system conditions.

***Main Messages*:**

• *MRCP has a fundamental role in the non-invasive detection of pancreatic ducts.*

• *In physiologic conditions pancreatic ducts are not always well detected on MRCP.*

• *Secretin injection causes temporary dilation of pancreatic ducts and thus improves MRCP detection.*

• *Secretin-stimulated MRCP may allow the assessment of the exocrine pancreatic reserve.*

• *Secretin increases the diagnostic capabilities of MRCP for evaluating pancreatic disorders.*

## Introduction

Endoscopic retrograde cholangiopancreatography (ERCP) is still considered the most sensitive and specific technique for the assessment of the pancreatic ductal system. However, ERCP is an invasive method that can potentially cause complications, though rarely severe. Another disadvantage is that it does not provide information on extra-ductal lesions and does not allow the visualisation of the obstructed segment in the event of total duct obstruction.

MR cholangiopancreatography (MRCP) is a non-invasive imaging technique that accurately detects the morphologic features of the pancreatic ducts. In several studies [1–4], MRCP demonstrated a good correlation with ERCP in the evaluation of disease and anatomic variants of the pancreatic ductal system. However, in physiological conditions the pancreatic ducts, particularly the side branches, are not always easily recognisable [2, 5].

Secretin is a peptide hormone produced by the intestinal mucosa, especially in the duodenum, and stimulates the fluid volume secreted by the exocrine pancreas. The increased volume causes temporary dilation of the pancreatic ducts and allows a better visualisation of them in MRCP; this condition can improve the detection and characterisation of pancreatic duct disorders [5–9].

More recently, secretin-enhanced MRCP protocols have been developed for a more complete assessment of the pancreatic ducts and glandular function, including monitoring of pancreatic flow dynamics and duodenal filling after pancreatic hormonal stimulation with secretin. Furthermore, some authors are also exploring the feasibility of this technique in the remnant pancreas after pancreatoduodenectomy and in the pancreatic graft [10–12].

In this pictorial review, we present our experience with secretin-stimulated MRCP (SS-MRCP) findings observed in patients with diagnosed or suspected pancreatic disease, focussing on the main clinical applications of this MR technique. All these clinical cases were carried out at a large university hospital specialising in hepato-biliary diseases.

## MR imaging technique

At our institution, MR imaging is performed with superconductive 1.5-T and 3.0-T devices (Signa HDx and GE-DISCOVERY MR750; GE Healthcare, Milwaukee, WI, USA) and phased-array multi-channel coils. All patients fast for at least 4–6 h prior to the examination and, 10 min before MRI, ingest a super-paramagnetic suspension (Lumirem® 100 ml, Guerbet) to suppress the signal intensity of overlapping fluid-containing organs. As an alternative for ferumoxsil suspensions, 5 ml of gadolinium-DTPA mixed in 75 ml of distilled water can be used to shorten the T2 time by acting as a negative T2 agent. Scopolamine methyl-bromide (Buscopan® 20 mg/ml, Boehringer Ingelheim) is intramuscularly administered immediately before starting the examination in order to avoid peristaltic artefacts.

The imaging protocol begins with axial, breath-hold, with and without fat-suppressed spoiled gradient-echo (SPGR) T1-weighted images followed by axial, respiratory-triggered, fat-suppressed, fast spin-echo (FSE) T2-weighted and/or axial, breath-hold, single-shot fast spin-echo (SSFSE) T2-weighted sequences.

Conventional MRCP is usually performed by using two techniques: respiratory-triggered, thin-collimation (2.4 mm thickness/-1.2 mm) three-dimensional FRFSE T2w sequences in the coronal plane and breath-hold, thick-slab (40-60 mm), single-shot FSE T2w sequences performed in the coronal and coronal oblique projections.

After the acquisition of the first image, secretin (Secrelux®, Sanochemia; 1 cU/kg body/weight) is injected intravenously in order to stimulate the pancreas to produce exocrine secretion. Secretin is administered via a slow intravenous injection for 1 min to avoid the potential adverse effect of abdominal pain that can occur with a bolus injection. SS-MRCP is performed using a coronal breath-hold, thick-slab, SSFSE T2w sequence covering the pancreas and adjacent small bowel; single-slice image acquisition is repeated every 30 s up to 15 min. Secretin has a very favourable safety and tolerability profile causing nausea, flushing or vomiting in only 0.5 % of patients. The only contraindication to the use of secretin documented by the manufacturer is acute pancreatitis. However in our institution secretin is used in patients with mild acute pancreatitis but is avoided in those with severe pancreatitis. The administration of secretin is therefore usually very well tolerated.

The peak effect of intravenous secretin administration is usually observed at 3-5 min after injection. At this time the calibre of the main pancreatic duct can increase by 1 mm or more compared with the baseline measurement and the side branches may become visible and be helpful for the diagnosis [13].

**Fig. 1:**
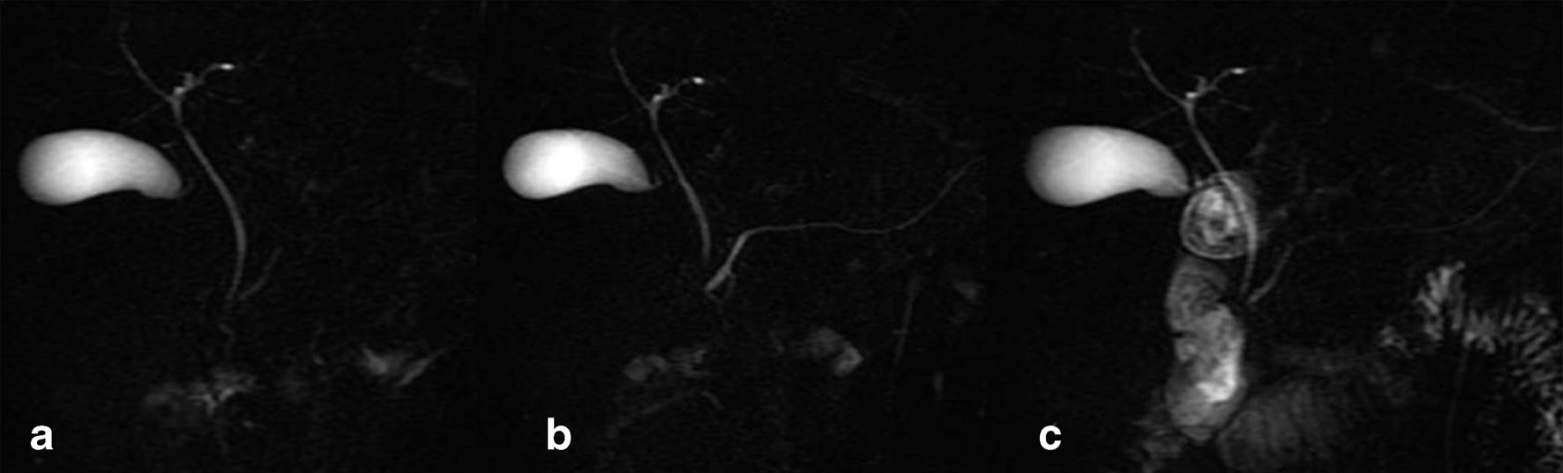
**a**–**c** Normal findings. MRCP performed before secretin injection shows a normal main pancreatic duct (**a**), which demonstrates a regular dilation 3 min after secretin stimulation (**b**) and normal duodenal filling beyond the genu inferius after 15 min (**c**)

A functional evaluation of the exocrine pancreatic reserve can be performed by a semi-quantitative image analysis of the duodenal filling on the basis of pancreatic secretion after secretin stimulation. The pancreatic fluid outflow is visually analysed on the image time series by the observers. The duodenal filling volume can be graded according to the scale (0–3) derived by Matos et al. [2] (Fig. 1).

## Clinical applications

### Anatomic variants

SS-MRCP improves the visualisation of the full length of the main pancreatic duct resulting in higher sensitivity and specificity in detecting congenital anomalies/malformations and in confirming or excluding entities such as pancreas divisum (PD) and anomalous pancreatico-biliary junction (APBJ).

PD is the most common congenital anatomical variant of pancreatic ductal development (about 15 %–20 % of patients with unexplained pancreatitis have been found to have PD, whereas only 5 %–10 % of the general population present such malformation) caused by the lack of fusion between the ventral and dorsal pancreatic ducts during the 6th-8th week of gestation. Because the major part of the pancreatic secretion must flow through the minor pancreatic duct, this could predispose to obstructive pancreatopathy causing pancreatitis and pancreatic-type pain or even the development of severe chronic pancreatitis [14, 15].

Another anatomic variant, called “incomplete pancreas divisum” (IPD), shares the feature of excretion of the major fraction of pancreatic secretions via the dorsal duct orifice. IPD is less associated with pancreatitis than complete PD, since high pressure in the dorsal pancreatic duct system can decrease via a connection to the ventral duct [12, 15, 16].

**Fig. 2:**
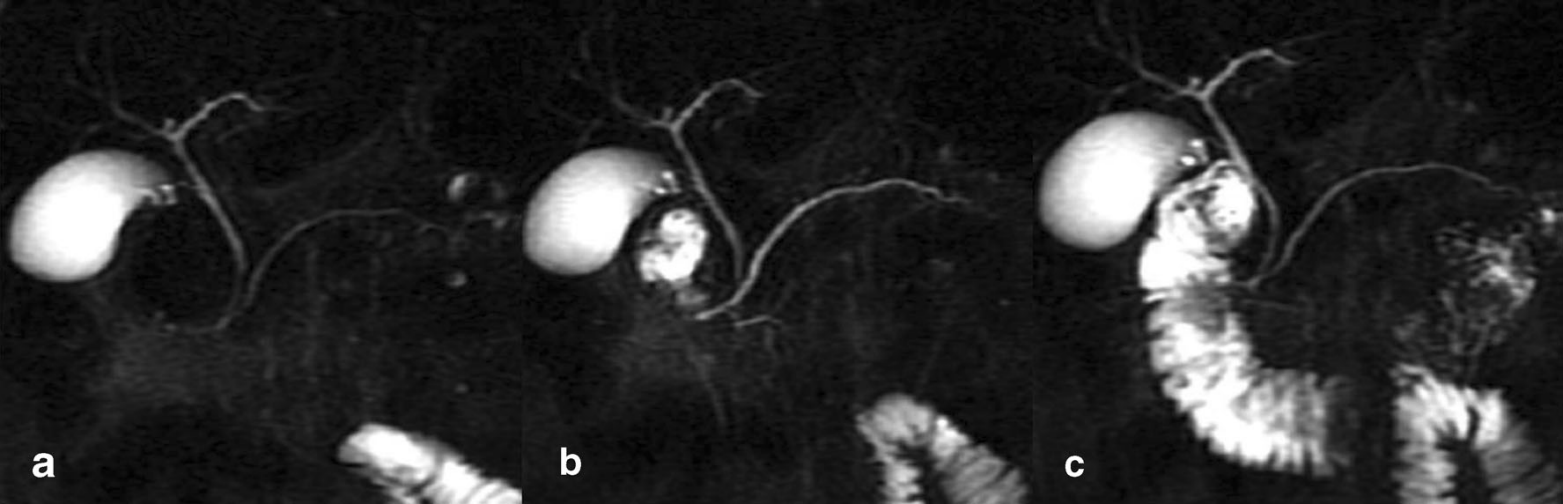
**a**–**c** Complete pancreas divisum. Before secretin stimulation, MRCP (**a**) reveals only the dorsal pancreatic duct. MRCP obtained 3 min after secretin injection (**b**) demonstrates both the ventral and the dorsal pancreatic duct, without connection between them and prompt outflow of pancreatic secretion via the minor papilla, corresponding to pancreas divisum with dominant dorsal duct. MRCP obtained 15 min after secretin injection (**c**) shows normal duodenal filling beyond the genu inferius

**Fig. 3:**
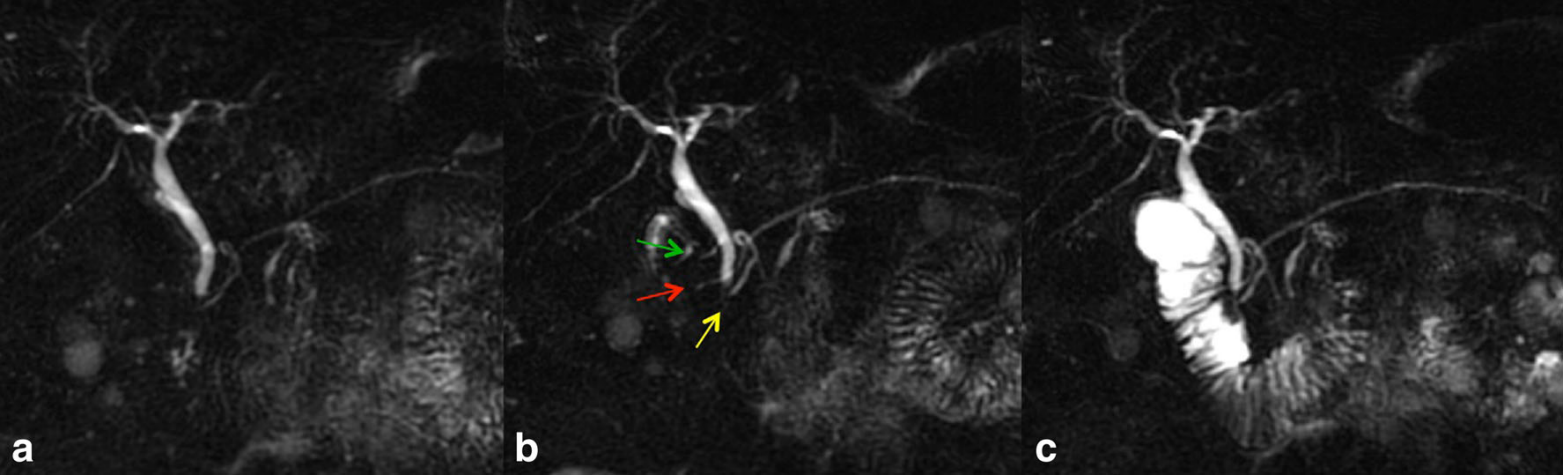
**a**–**c** Incomplete pancreas divisum. Before secretin-stimulation, MRCP (**a**) shows both the dorsal and the ventral pancreatic duct connected between them and the major and minor papilla. After secretin injection (**b**, **c**) two other secondary ducts and another cranial accessory papilla are visualised (arrows), with initial outflow of pancreatic secretion via the accessory papilla

SS-MRCP and MRI can serve as a comprehensive diagnostic tool without radiation for the diagnosis of PD (Fig. 2) or IPD (Fig. 3), whereas the ERCP can be reserved for clinical conditions that require interventional procedures for therapeutic purposes [17].

APBJ is a rare congenital anomaly in which the pancreatic and biliary ducts are joined outside the duodenal wall [15], which may be diagnosed by distention of the gallbladder during SS-MRCP.

Santorinicele is a cystic dilation of the distal dorsal duct, just proximal to the minor papilla, and is believed to result from a combination of relative obstruction and weakness of the distal duct wall, either acquired or congenital. Santorinicele has been suggested as a possible cause of relative stenosis of the accessory papilla that may become clinically relevant when it occurs in association with PD or IPD, resulting in recurrent episodes of acute pancreatitis.

According to some authors, SS-MRCP is effective in diagnosing the presence of Santorinicele in patients affected by unexplained recurrent episodes of acute pancreatitis, who might benefit from endoscopic sphinterotomy of the minor papilla [15, 18].

### Chronic pancreatitis

Chronic pancreatitis is defined as a continuing inflammatory destruction of pancreatic tissue that results in irreversible damage to the parenchyma and ductal system, causing loss of the exocrine and/or endocrine function. Diagnosis is made by clinical history, testing of the pancreatic exocrine function and imaging.

As to the evaluation of the pancreatic exocrine function, the most sensitive diagnostic tool to detect chronic pancreatitis at its earliest stage is the secretin-stimulated endoscopic pancreatic function testing (ePFT): pancreatic exocrine reserve is measured by duodenal aspiration after direct stimulation of the gland. The disadvantages of this test include its invasiveness, long procedure time and sedation of the patient during the procedure. A reliable non-invasive alternative technique is the SS-MRCP, where imaging findings (reduced duodenal filling grade and reduced increase in pancreatic duct caliber) are comparable to the results of ePFT [19].

MRI allows the evaluation of early fibrotic changes, glandular volume depletion or atrophy, and pancreatic progressive enhancement that peaks on the portal-venous or interstitial phase.

However, parenchymal changes might be preceded by ductal changes in chronic pancreatitis.

**Fig. 4:**
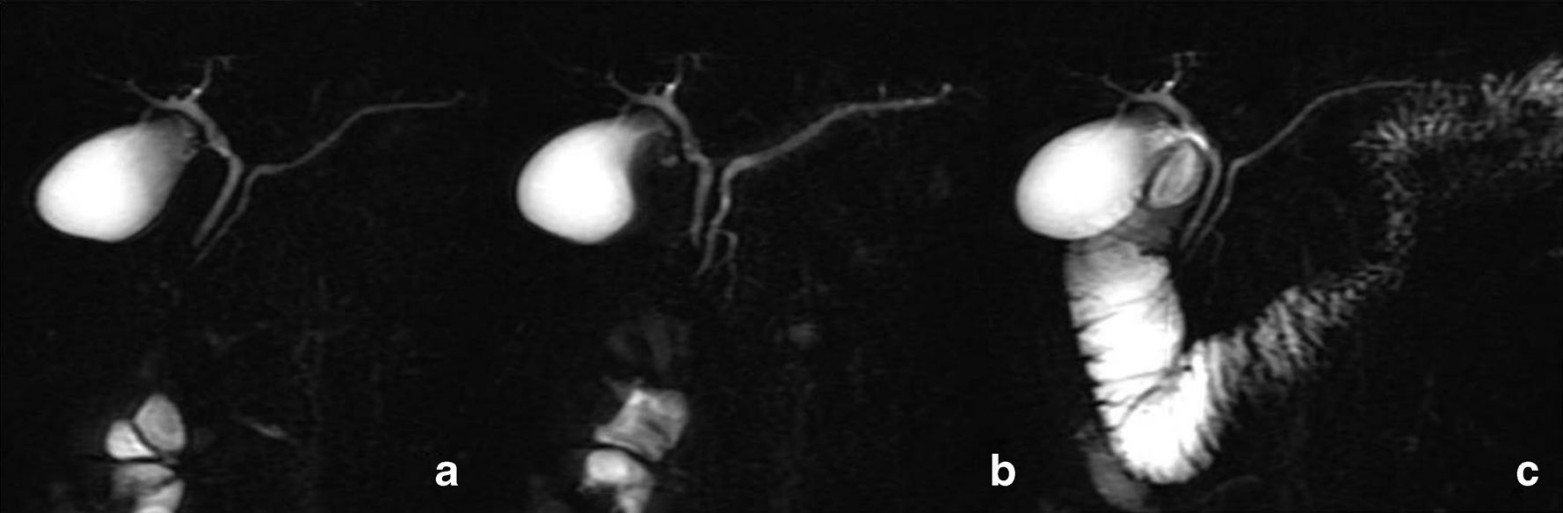
**a**–**c** Mild chronic pancreatitis. MRCP obtained before secretin injection (**a**) shows a normal main pancreatic duct. Three minutes after secretin stimulation (**b**), MRCP reveals side branch dilation, particularly at the level of the head and tail diagnosed as mild chronic pancreatitis. MRCP obtained 15 min after secretin injection (**c**) demonstrates normal duodenal filling beyond the genu inferius, interpreted as preserved pancreatic exocrine reserve

MRCP findings in early chronic pancreatitis often display a normal main pancreatic duct because of the underestimation of ductal size. Some investigators reported that patients with abnormal MR imaging findings but normal MRCP might benefit from dynamic SS-MRCP. SS-MRCP may reveal ductal abnormalities otherwise not detected on MRCP alone because of improved visualisation, and it provides images comparable to ERCP according to the Cambridge classification [20]. SS-MRCP has been reported to show ductal changes such as an increased number of side branch ectasia, irregular morphology of the pancreatic duct and/or decreased pancreatic duct compliance after secretin stimulation (Fig. 4).

**Fig. 5:**
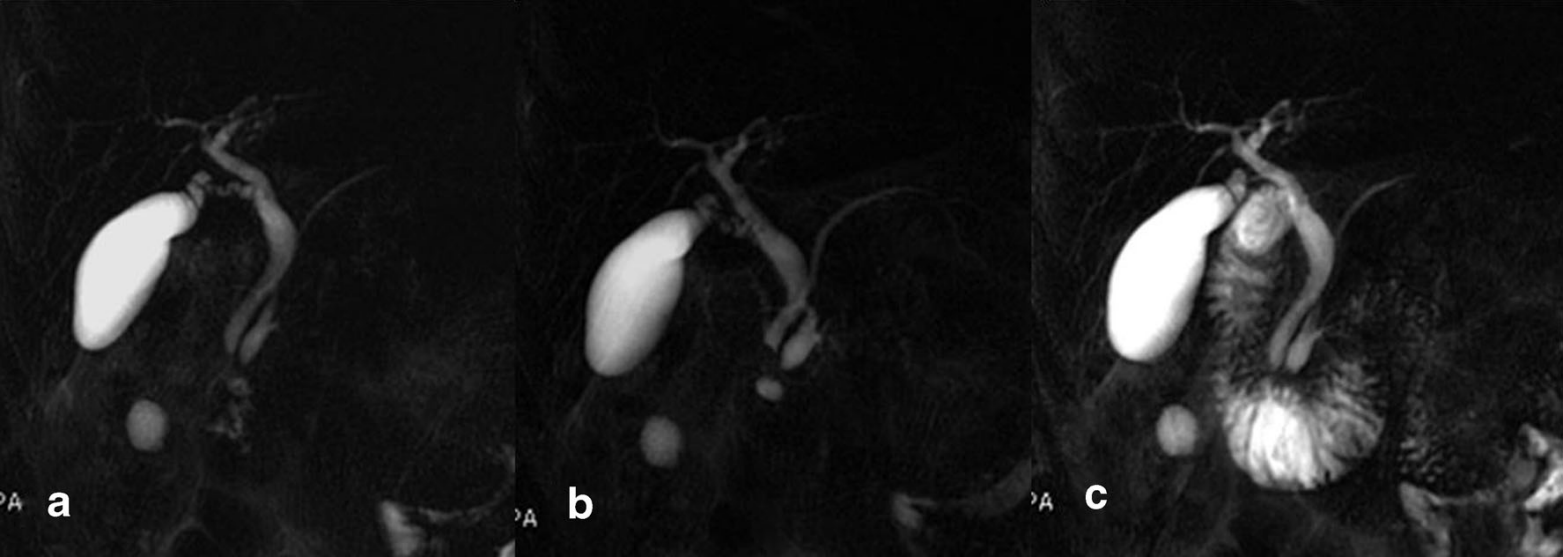
**a**–**c** Inflammatory ampullary stenosis. MRCP obtained before secretin administration (**a**) reveals distal common bile duct stenosis, with consequent dilatation of extra-hepatic bile ducts. The head portion of pancreatic duct is only slightly dilated. MRCP obtained 3 min after secretin injection (**b**) shows increasing diameter of the pancreatic duct and after 15 min (**c**) demonstrates duodenal filling up to genu inferius (grade 2), interpreted as reduced exocrine pancreatic reserve. Endoscopic evaluation with papillary biopsy confirmed the diagnosis

Discrepancies between standard MRI/MRCP findings and pancreatic exocrine function exist. In a study by Balci et al., the severity of pancreatic duct and side branch changes were the same in both groups of normal and abnormal exocrine function values. A patient with normal pancreatic exocrine function may have abnormal pancreatic ductal changes consistent with chronic pancreatitis. Patients with chronic pancreatitis may have maintained the pancreatic exocrine function [19]. Any stricture or obstruction in the distal duct or ampulla can increase the distention of the upstream portion of the duct in response to secretin (Fig. 5). Because strictures are often found in patients with chronic pancreatitis, ductal distention in such cases can be falsely reassuring. On the other hand, ductal distention in response to secretin administration is not seen in normal patients who have undergone prior pancreatic sphincterotomy because of the lack of pressure at the orifice [16]. Though the technique does not always allow a correct evaluation of the exocrine pancreatic function, it must be stressed again that SS-MRCP is a non-invasive technique and its results are comparable to those of ePFT.

### Focal pancreatic lesions

The detection of a focal enlargement or distortion of the normal contour of the pancreas is a common finding for radiologists who treat pancreatic diseases. Patients with such focal enlargements of the pancreas will have a conventional pancreatic carcinoma (PC) even if pathognomonic features of pancreatic carcinomas are still lacking, while a small percentage of them may have an inflammatory pancreatic mass (IPM).

Although most patients with IPM have somewhat characteristic histories, such as alcohol abuse, previous episodes of pancreatitis and recurrent abdominal pain for at least 2 years, the differential diagnosis between IPM and PC remains a clinical dilemma. Moreover, if the IPM is caused by the autoimmune pancreatitis developed in the proximal pancreatic portion and associated with obstruction of the common bile duct and/or main pancreatic duct, the clinical and radiological diagnosis is even more difficult. As a consequence, patients with the focal type of autoimmune pancreatitis often undergo surgery [21].

MR imaging with MRCP is superior to both MR imaging alone and CT in differentiating focal autoimmune pancreatitis or another form of inflammatory pancreatic disease from carcinomas, considering that focal IPM tends to have an irregular but not obstructed main pancreatic duct, which tends to penetrate the mass after secretin administration. Moreover, in inflammatory masses the narrowing of the dilated duct tends to be multiple and gradual, while in pancreatic cancer there is usually a single abrupt interruption [22].

**Fig. 6:**
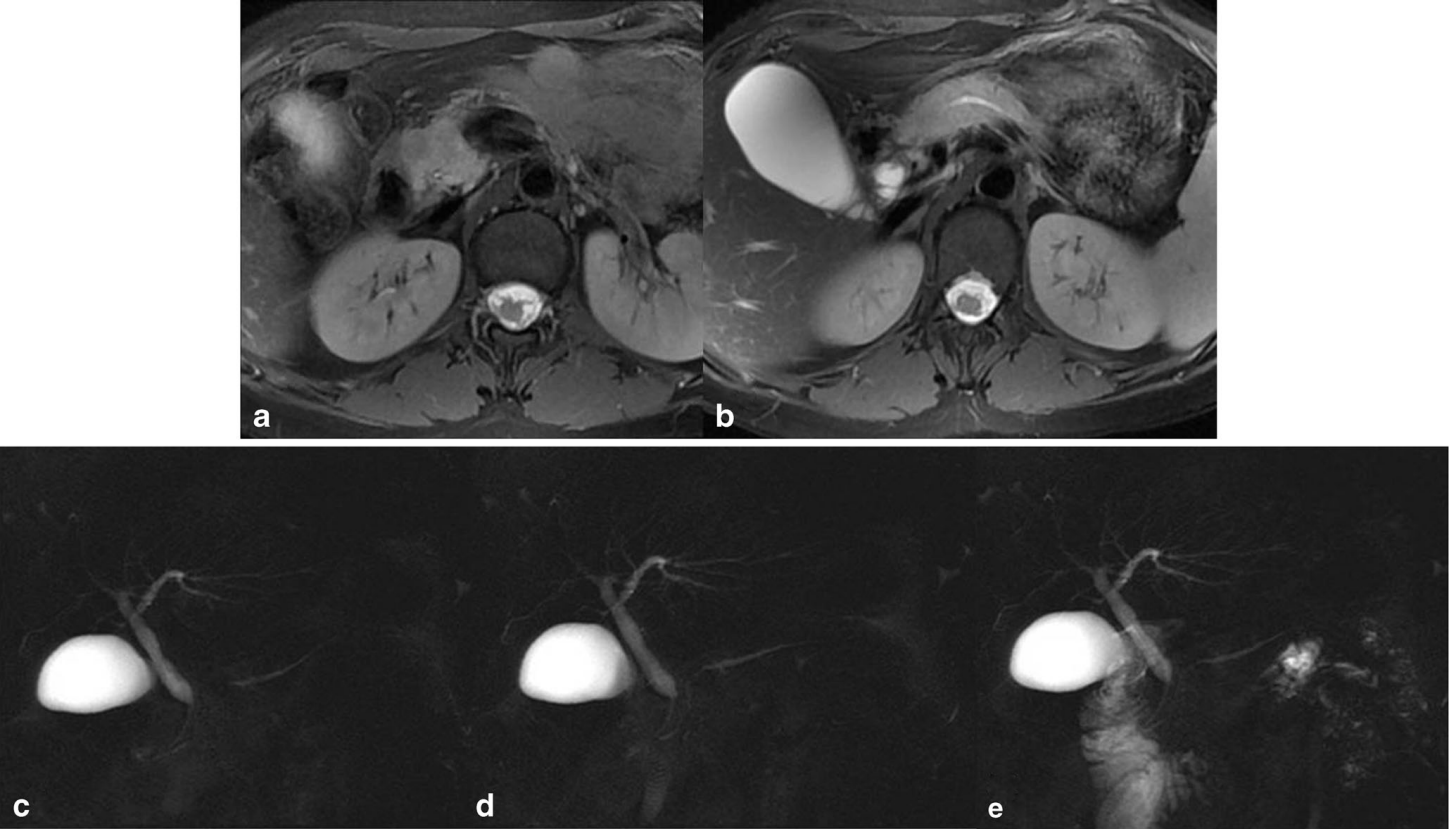
**a**–**e** Autoimmune pancreatitis. Axial T2-weighted images (**a**, **b**) show loss of the normal pancreatic lobulations at the level of the head and focal obliteration of the pancreatic duct, better demonstrated on conventional MRCP (**c**). After secretin administration (**d**, **e**) the stricture detected in basal phase is solved (duct-penetrating sign)

**Fig. 7:**
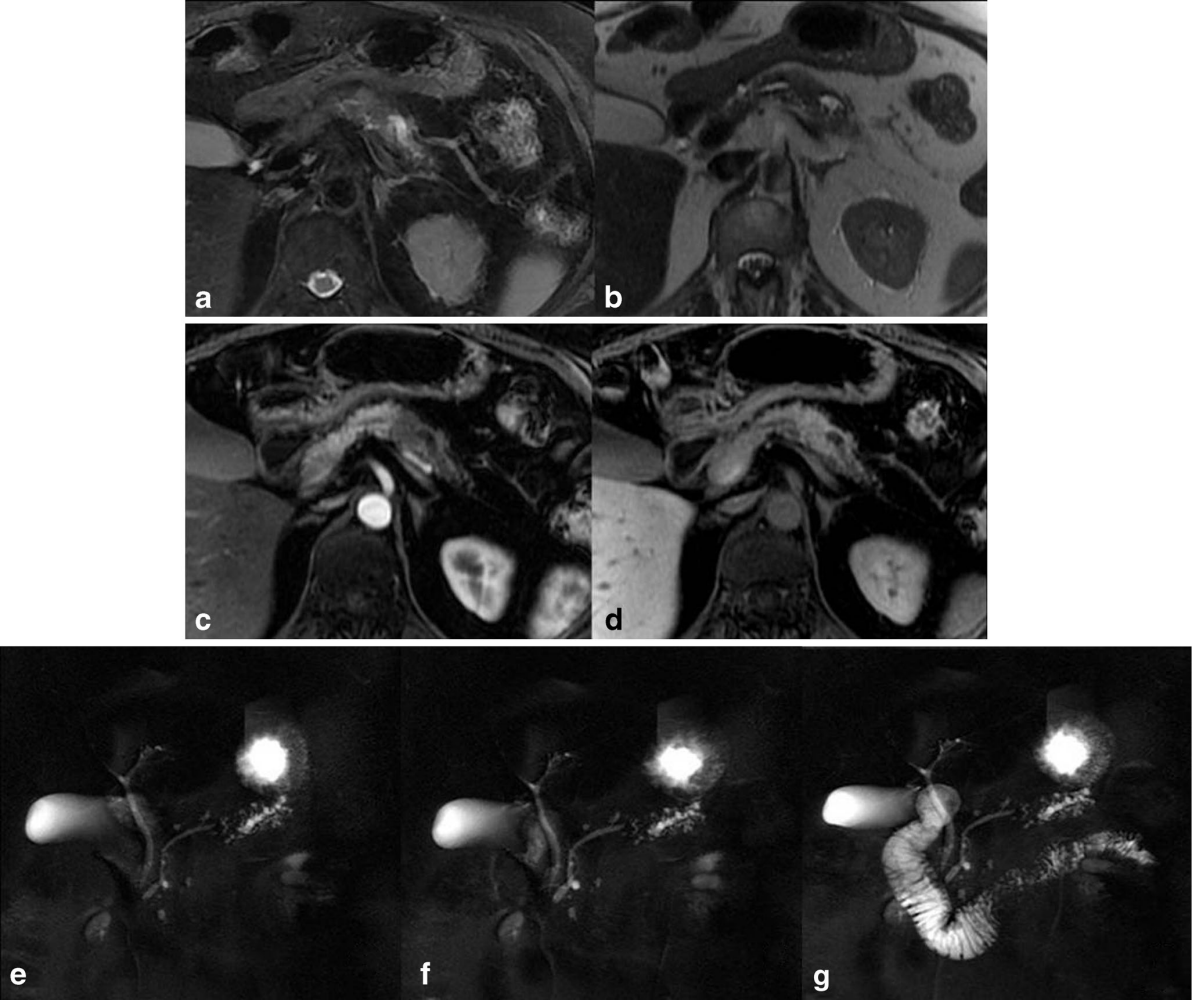
**a**–**g** Ductal adenocarcinoma. Axial T2-weighted (**a**, **b**) and axial post-contrast T1-weighted images (**c**, **d**) show a small lesion at the level of the pancreatic body with an abrupt cut-off of the main pancreatic duct, better visualised on MRCP (**e**). After secretin stimulation (**f**, **g**) there is a persistent stenosis, without the duct-penetrating sign

The so-called “duct-penetrating sign”, defined as the presence of a stenosis of the main pancreatic duct coursing through the mass, which enlarges after secretin administration, has a sensitivity of 85 % and a specificity of 96 % for the distinction between focal inflammatory mass and pancreatic cancer (Figs. 6 and 7).

Carbognin et al. corroborate the importance of the SS-MRCP sequence by showing a strong correlation between the duct-penetrating sign and the mass-forming of autoimmune pancreatitis and an association between the obstruction of the main pancreatic duct and pancreatic carcinoma. These characteristics improve the IPM diagnosis if associated with other MR image findings, such as delayed pancreatic enhancement and a capsule-like smooth rim of the mass (an uncommon but pathognomonic detail useful in differentiating IPM from carcinoma) [23].

### Cystic pancreatic lesions

Pancreatic cystic neoplasms (PCNs) are common lesions, which in clinical practice are often detected incidentally. Surgical indications for PCN are limited to symptomatic lesions and to lesions that are considered malignant or with a potentially high risk of malignancy on the basis of the pre-operative diagnostic assessment.

Main duct IPMN (MD-IPMN) is more frequently associated with this malignant transformation than is branch duct IPMN (BD-IPMN). Because of the high malignant potential, surgical resection is in general recommended for MD-IPMN, whereas BD-IPMN management is more conservative with imaging surveillance, especially in asymptomatic patients with cysts measuring <3 cm and without features considered worrisome for malignancy (e.g. mural nodules; thick, enhancing walls) [24].

**Fig. 8:**
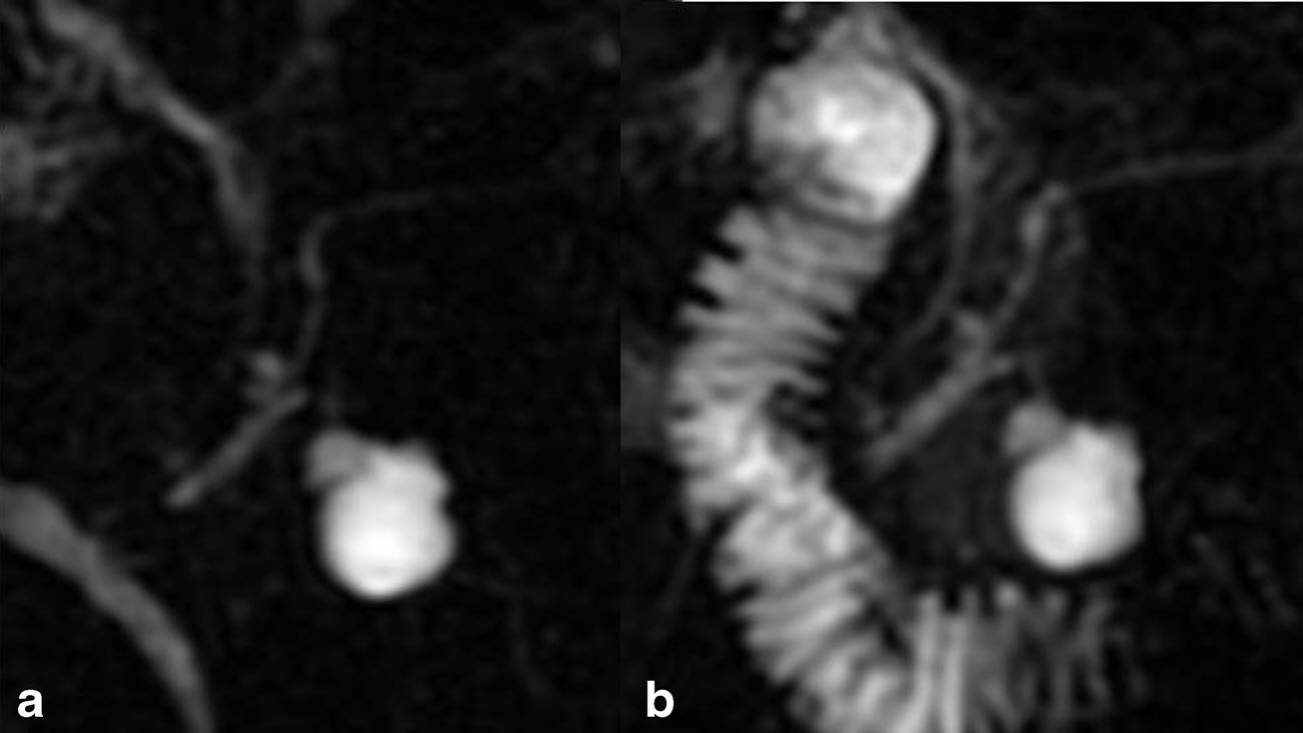
**a**, **b** Branch duct-type intraductal papillary mucinous tumour in the uncinate process of the pancreas. Conventional MRCP (**a**) reveals a bilocular cystic lesion without a sure connection to the main pancreatic duct. After secretin injection (**b**) the pancreatic ductal system is better delineated and it is also possible to appreciate the relationship between the lesion and the Wirsung duct

BD-IPMN may show a more segmental cystic appearance that can mimic the appearance of other cystic neoplasms of the pancreas, such as non-neoplastic cysts. Thus, the main diagnostic challenge is to accurately distinguish IPMN from benign cystic neoplasms of the pancreas such as serous cystadenomas. In this scenario, the visualisation of the communication between the BD-IPMN and the pancreatic duct system is a key feature that allows us to distinguish IPMNs from cystic lesions of other aetiologies. MRCP uses the almost stationary fluid in the biliary and pancreatic ductal system as an intrinsic contrast medium that can be improved in the detailed evaluation of the biliary and pancreatic ductal anatomy of IPMN by administration of secretin [2, 12, 23]. Secretin allows for better visualisation of the ducts at MRCP and improves the visibility of a communication between the main pancreatic duct and the cystic lesion (Fig. 8) [23]. Moreover, secretin increases the cyst size or signal intensity and permits better assessment of ductal irregularities.

However, the diagnostic utility of secretin in the diagnosis of pancreatic cysts remains debated in part because there have not been controlled studies assessing the added diagnostic value of secretin [12, 25, 26].

### Post-operative findings

#### Pancreato-jejunal anastomosis

Pancreatico-duodenectomy is among the most common surgeries performed for pancreatic pathology.

Although in-hospital mortality is now just a fraction of what it once was, post-operative complication occurs in approximately 30–50 %.

While the bilio-enteric and entero-enteric strictures can be addressed through minimally invasive endoscopic or radiographic means, management of a pancreaticojejunal anastomosis is challenging in that the altered anatomy of the post-pancreaticoduodenectomy reconstruction often precludes successful employment of such techniques.

The patency of pancreaticojejunostomy anastomosis is of paramount importance in conserving the exocrine function of the pancreas in patients who have undergone partial pancreatectomy.

Indeed, permanent endocrine and exocrine dysfunctions usually manifest within the first month of recovery, but a minor number of cases have been described as a long-term complication of pancreaticoduodenectomy, demonstrating that pancreatic insufficiency and atrophy of the pancreatic parenchyma are related to both post-surgical alteration of pancreatic neurohormonal stimulator mechanisms and stenosis of the pancreatojejunal anastomosis.

Early diagnosis is, therefore, needed to allow for immediate endoscopic or surgical treatment.

Secretin MRCP proved to be a useful tool for diagnosis as it allows for a ‘functional’ test of the remnant pancreas, and it is more discriminating for ductal stenosis than is traditional static MRCP [27].

**Fig. 9:**
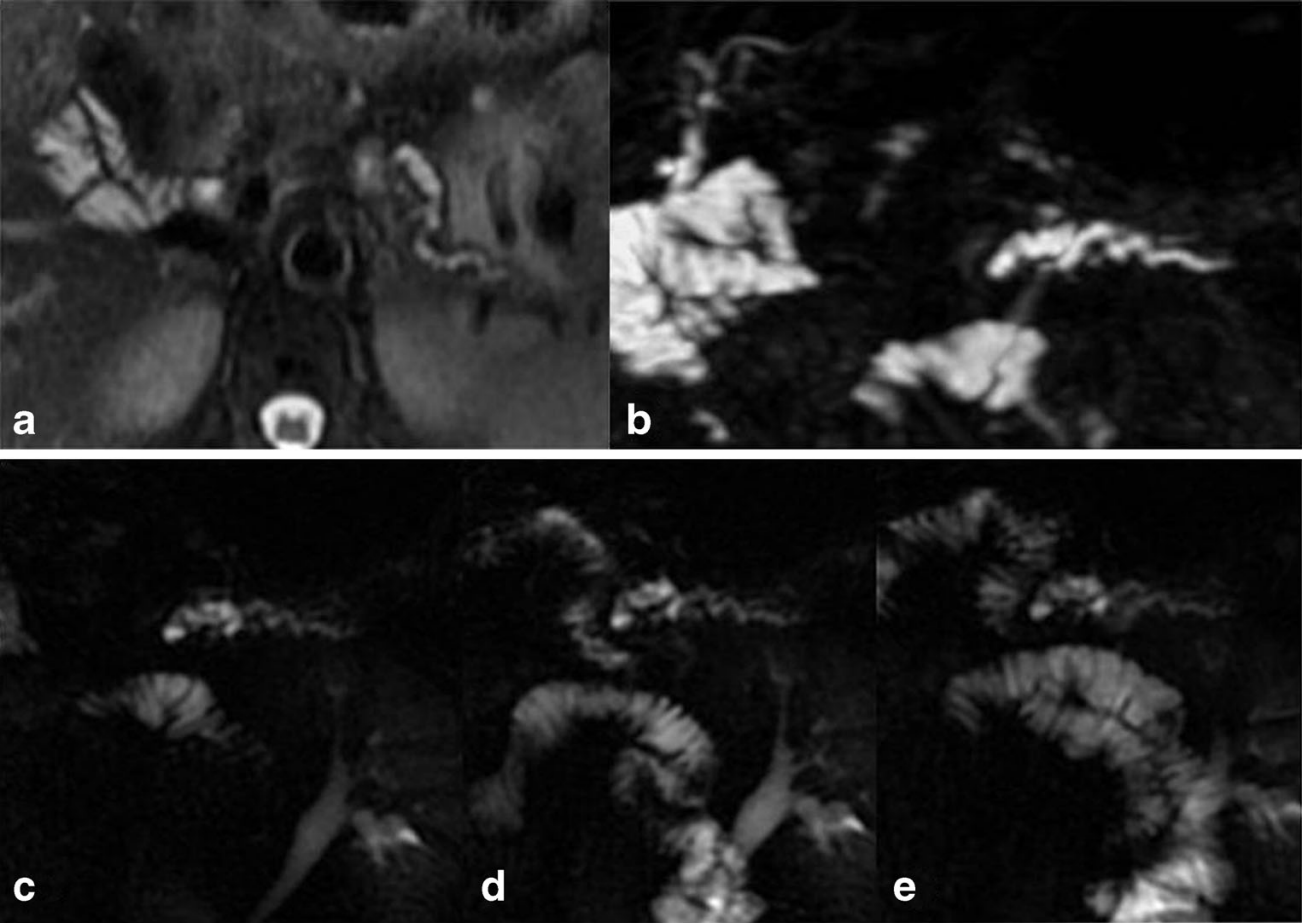
**a**–**e** Pancreaticoduodenectomy. Axial T2w (**a**) and MIP (**b**) images show the pancreatic body and tail with slight and irregular dilation of the main pancreatic duct. Secretin-stimulated MRCP (**c**–**e**) exhibits patency of anastomotis with normal filling of jejunal loops well

The addition of secretin significantly improves the visualisation of the anastomotic site and allows the evaluation of the post-secretin jejunal filling, which indicates relative preservation of pancreatic function (Fig. 9) [28].

#### Pancreatic transplantation

The aim of pancreas transplantation is to restore normoglycaemia, curing diabetes and limiting the progression of complications associated with diabetes. In the majority of cases, pancreas transplantation is performed in individuals with type 1 diabetes that have end-stage renal disease, usually with uraemia, retinopathy, progressive neuropathy and hypoglycaemic unawareness. This procedure enables a total restoration of endogenous secretion of insulin as well as regression or stabilisation of the degenerative complications of diabetes.

Despite the improvement in both surgical techniques and post-operative management, there are significant complications following pancreas transplantation, particularly graft rejection. Graft loss through rejection occurs as a result of alloimmunity or autoimmune recurrence; however, the latter remains difficult to determine. Rejection rates are around 5 %–25 % depending on what immunosuppressive regime is used.

The diagnosis of acute rejection can be established using transplanted kidneys as surrogate markers if they are from the same donor or direct biopsy of the pancreas in response to changes in biochemical markers or clinical presentation; however, biopsy is frequently accompanied by complications, such as inflammation of the gland, fistula of the main pancreatic duct and abdominal bleeding.

Various authors have tested MR imaging as a possible diagnostic non-invasive tool in the diagnosis of pancreatic graft rejection. Allograft oedema due to acute rejection can be recognised by means of increased T2 signal intensity; however, other causes of increased T2 signal include acute pancreatitis and ischaemia. There may be a substantial difference in enhancement between rejecting and normal pancreas allografts; however, this finding is only moderately specific.

**Fig. 10:**
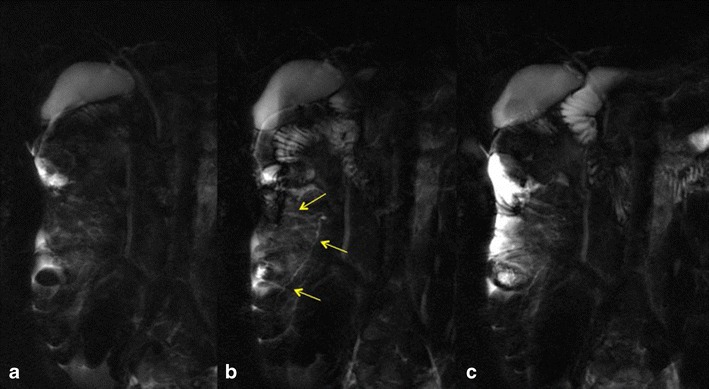
**a**–**e** Pancreatic transplant. On MRCP before secretin administration (**a**) the main pancreatic duct of the graft is not appreciable; however after secretin injection (**b**, **c**) it is possible to recognise both the Wirsung duct (arrows) and the normal duodenal-jejunal graft filling

Dynamic MR pancreatography after secretin stimulation has been shown to be a reliable diagnostic tool in the evaluation of functional status of the pancreatic transplants in patients undergoing isolated or combined kidney–pancreas transplantation [11]. After secretin administration, both variations in the diameter of the main pancreatic duct and graft fluid outflow can be used to demonstrate the exocrine function of the allograft (Fig. 10).

### Asymptomatic elevation of serum lipase and amylase

Chronic asymptomatic pancreatic hyperenzymaemia (also called “Gullo’s syndrome”) [29, 30] is a persistent abnormal increase in the serum concentrations of the pancreatic enzymes without pancreatic symptoms and evidence at imaging of pancreatic diseases. The medical need in these patients is to differentiate undiagnosed pancreatic disease and other extra-pancreatic conditions that may induce an increase in serum concentrations of amylase and lipase, including chronic viral hepatitis, renal failure, celiac disease, inflammatory bowel disease, hyperparathyroidism and neoplasms [21, 31]. Consistent evidence, however, indicates that asymptomatic subjects with increased pancreatic enzymes lasting for more than 2 years and accompanied by thoroughly negative imaging tests (pancreatic CT scan and/or MRCP) are indeed affected by benign pancreatic hyperenzymaemia [30].

SS-MRCP is the most appropriate imaging technique for investigating patients with chronic asymptomatic pancreatic hyperenzymaemia [21, 31]. The most frequent abnormalities diagnosed on secretin-enhanced MRCP images were diffusely dilated side branches (25.6 %), diffuse main pancreatic duct dilation and delayed emptying of the main pancreatic duct (14.4 %) [21]. A possible explanation for these alterations may be a delayed emptying of the pancreatic outflow, not able to trigger pancreatitis but sufficient to induce a disorder of the normal pancreatic intracellular exocytosis process and, therefore, a leakage of pancreatic enzymes into the blood through the basolateral membrane of the acinar cell.

In a study by Donati et al. [32] in 80 asymptomatic patients with persistent (for at least 6 months) non-specific serum hyperamylasaemia and hyperlipasaemia, conventional MRCP showed pancreatic abnormalities only in 50 % of patients, while, after secretin stimulation, the percentage of detection of pancreatic alterations was increased by 27.5 %. SS-MRCP may represent the best noninvasive diagnostic technique since it gives morphological and functional information on the pancreas, which has been found to be abnormal in 68.7 % of patients.

### Pancreatic trauma

Pancreatic injury in the setting of blunt abdominal trauma is uncommon, with reported incidence ranging from approximately 2 % to 12 %. The associated mortality is considerable, however, and may be as high as 30 % to 50 %, largely secondary to concomitant injuries.

Elevated serum amylase may be present, but the clinical presentation of pancreatic injury is variable and nonspecific. Blunt pancreatic injuries occur more commonly in the body of the gland, accounting for two-thirds of cases, and are typically caused by a crushing impact against the vertebral column.

Because the main cause for morbidity and mortality is disruption of the MPD, assessment for ductal injuries is critical [32].

Disruption was defined as a discontinuity in the duct or, better, the visualisation of increasing amounts of fluid outside the duct or proximal small bowel after secretin stimulation. Apparent duct discontinuity in patients with pancreatitis can have a number of causes, including compression of the duct by fibrosis or oedema, strictures, stones or adjacent magnetic susceptibility artefacts from gas, haemosiderin or metal [32].

The causes of pancreatic duct disruption also include acute pancreatitis, chronic pancreatitis and surgery [33].

Persistent disruption can result in fluid collections, ascites or fistulas and has a significant impact on the clinical course. Small side-branch disruptions can heal without long-term sequelae, but main-duct disruption can result in strictures, secondary recurrent acute pancreatitis, pancreatic atrophy, and eventually endocrine and exocrine insufficiency.

Current treatment options include surgery, endoscopic intervention or conservative management. Timely diagnosis of duct disruption, its location and the size of the leak are essential for choosing the appropriate treatment.

The clinical setting, ductal anatomy and parenchymal anatomy also affect the choice of therapy.

Cross-sectional imaging can be used to infer the presence and degree of disruption, although CT can be unreliable in the early diagnosis of pancreatic trauma.

Deep lacerations (involving greater than 50 % of the thickness of the pancreas) are predictive of ductal disruption and may be detected using T1-weighted post-contrast and T2-weighted sequences [32].

MRCP has been used in trauma patients to show peripancreatic fluid collections and the ductal anatomy, but only ERCP has been able to provide dynamic information as to whether there is continuing leakage from the duct. Even then, ERCP fails to reveal the disruption in as many as 25 % of instances and also carries the risk of introducing infection and the theoretic risk of exacerbating a leak by contrast injection at non-physiologic pressures.

Non-visualisation of a disruption is more likely to result in therapeutic failure, either surgical or endoscopic, and ERCP may fail to show the disruption because of duct obstruction proximal to it, and overfilling of the duct in pancreatic disruption is actively discouraged because of the risk of sepsis.

SS-MRCP is a non-invasive technique that can show: a) the whole pancreatic anatomy, both the parenchyma and the ducts; b) any peripancreatic fluid collections; c) disruption, including a leak, beyond an obstructed duct. SS-MRCP thus provides all the information available from CT plus a more complete assessment of the duct than often can be obtained on ERCP and dynamic information about ongoing leakage.

One caveat is that percutaneous drains should be clamped before SS-MRCP and the abdomen should be examined for any sinus tracts, which need to be monitored clinically or included in the images [33, 34].

## Conclusion

Secretin-enhanced MR cholangiopancreatography is a non-invasive imaging technique that usually improves the visualisation of the pancreatic ductal system. In particular, it can accurately depict the morphologic features and calibre modifications of the main pancreatic duct and side branches, and it can evaluate pancreatic flow dynamics through the duodenal papillae and duodenal filling after secretin stimulation.

Secretin increases the diagnostic capabilities of MRCP in various pancreatic diseases and can be indicated in patients with known or suspected anatomic variants, sphincter of Oddi dysfunction, chronic pancreatitis, main pancreatic duct stenosis, cystic pancreatic lesions, pancreatic trauma, chronic asymptomatic hyperenzymaemia and post-operative conditions such as pancreato-jejunal anastomosis and pancreatic transplantation.
